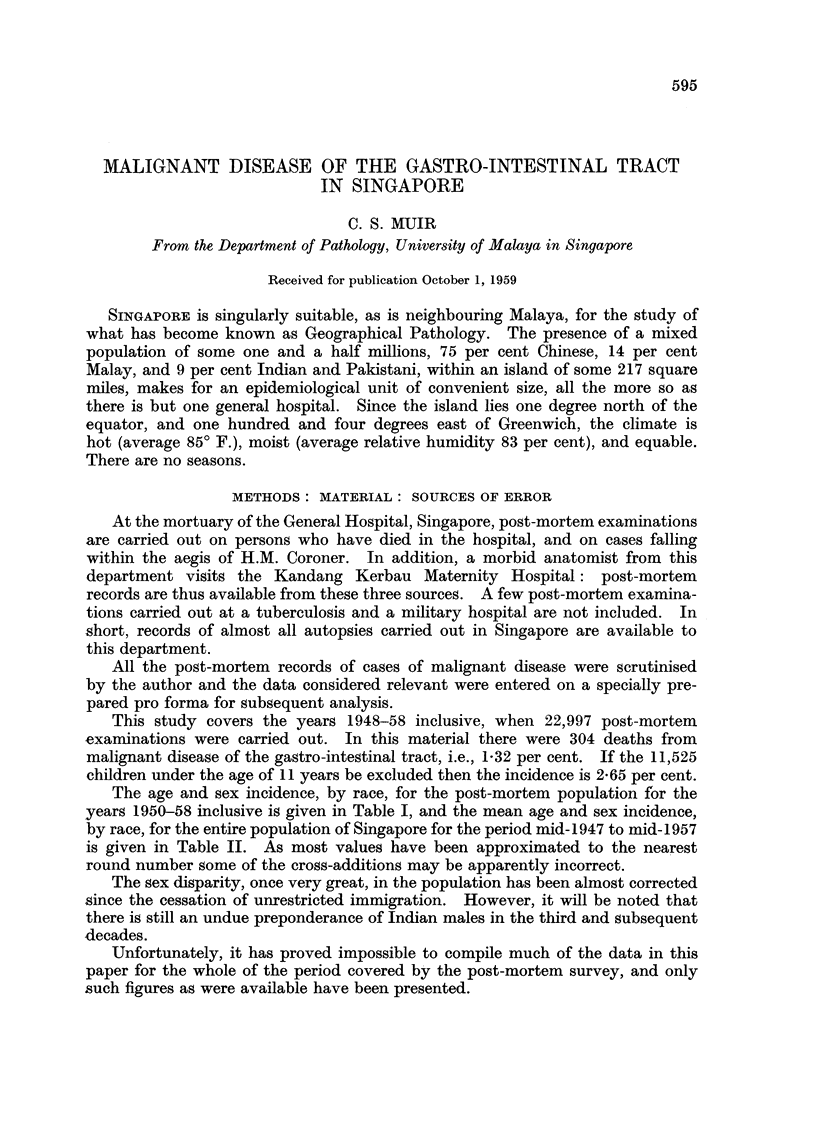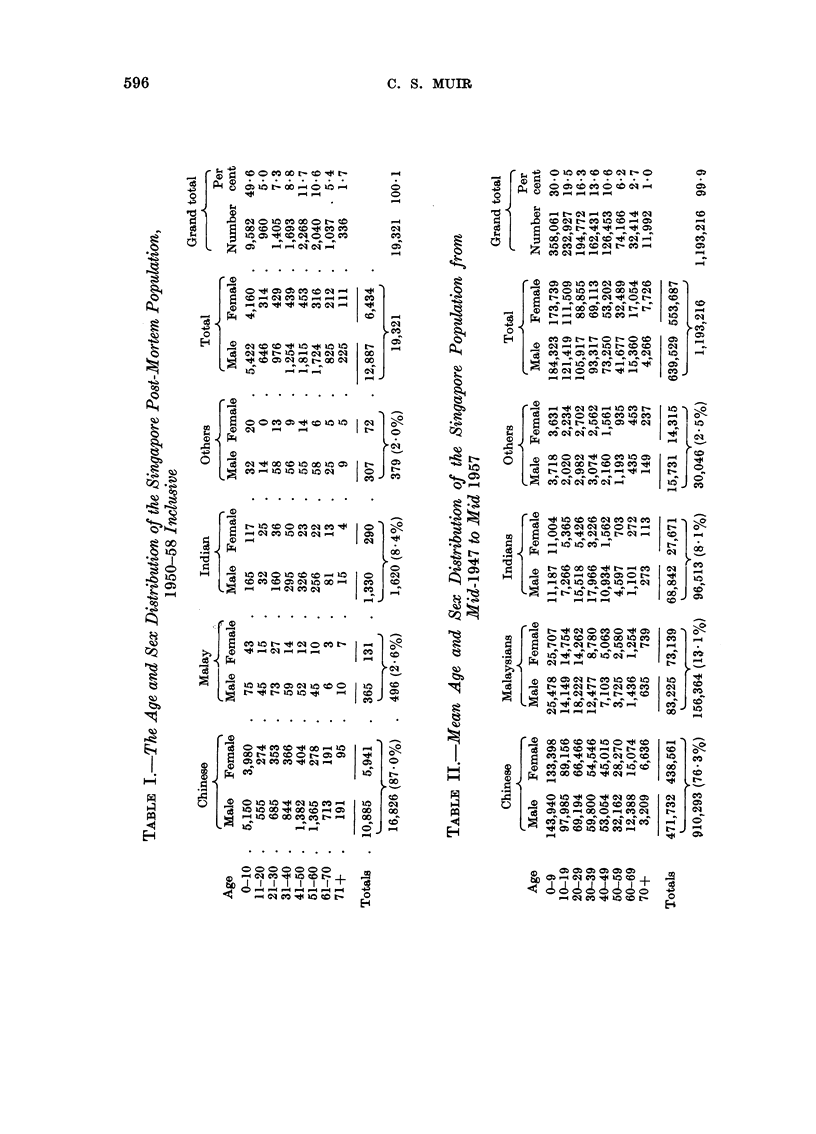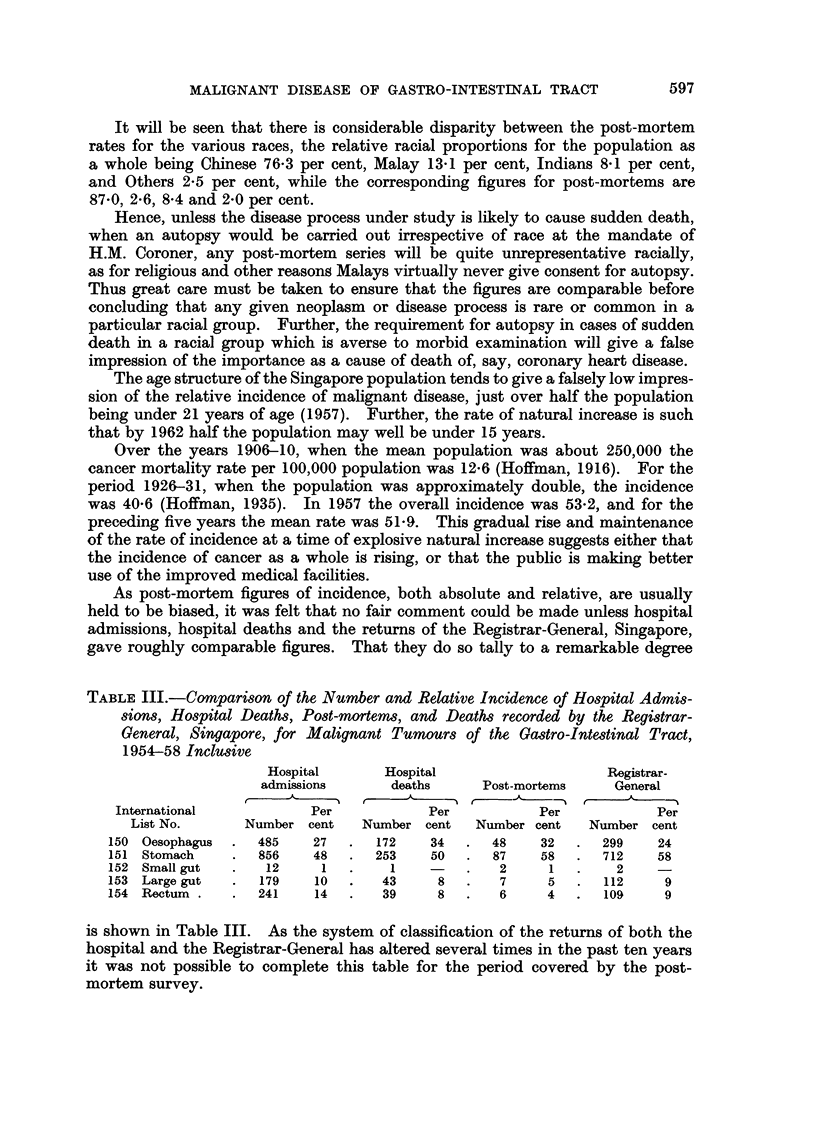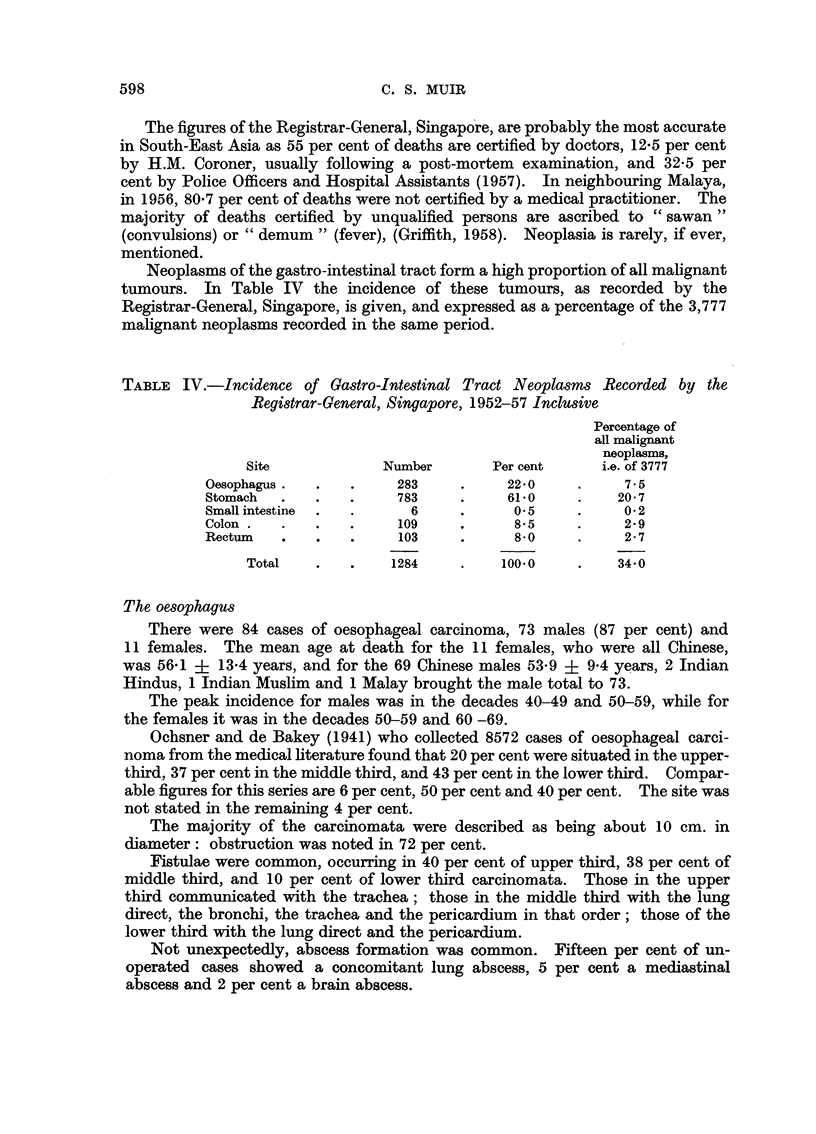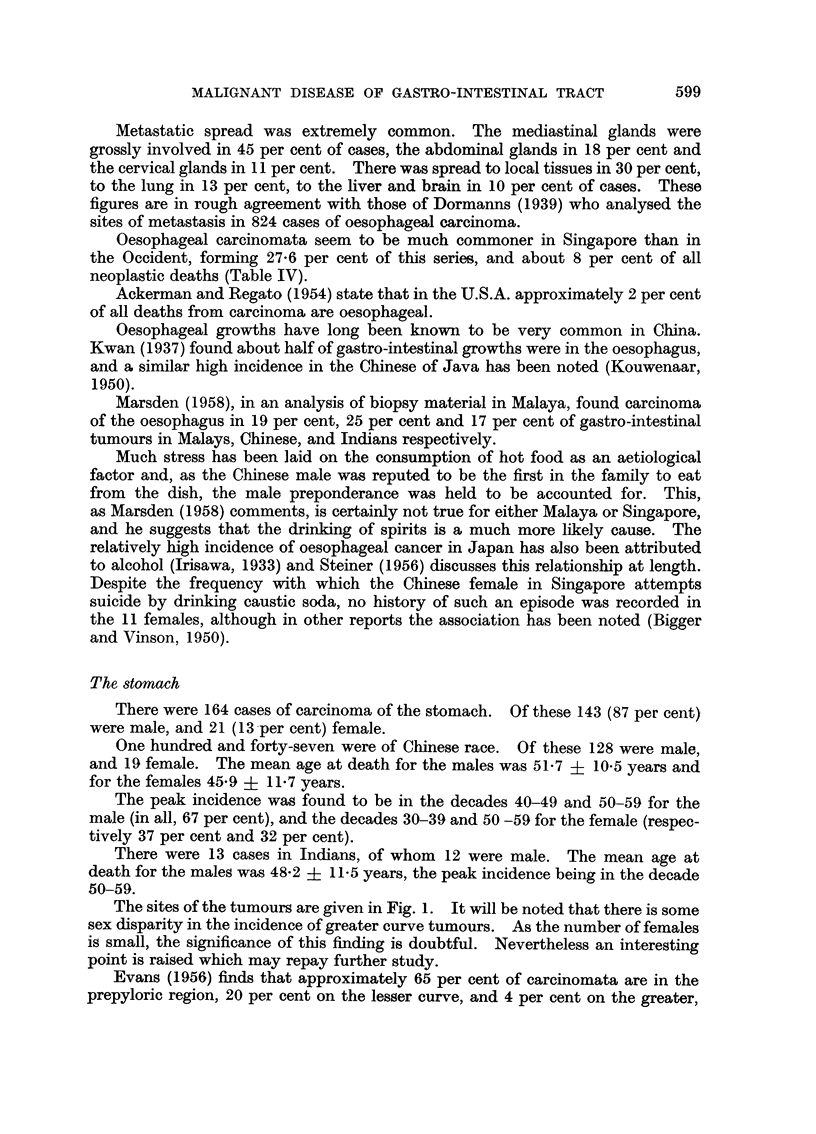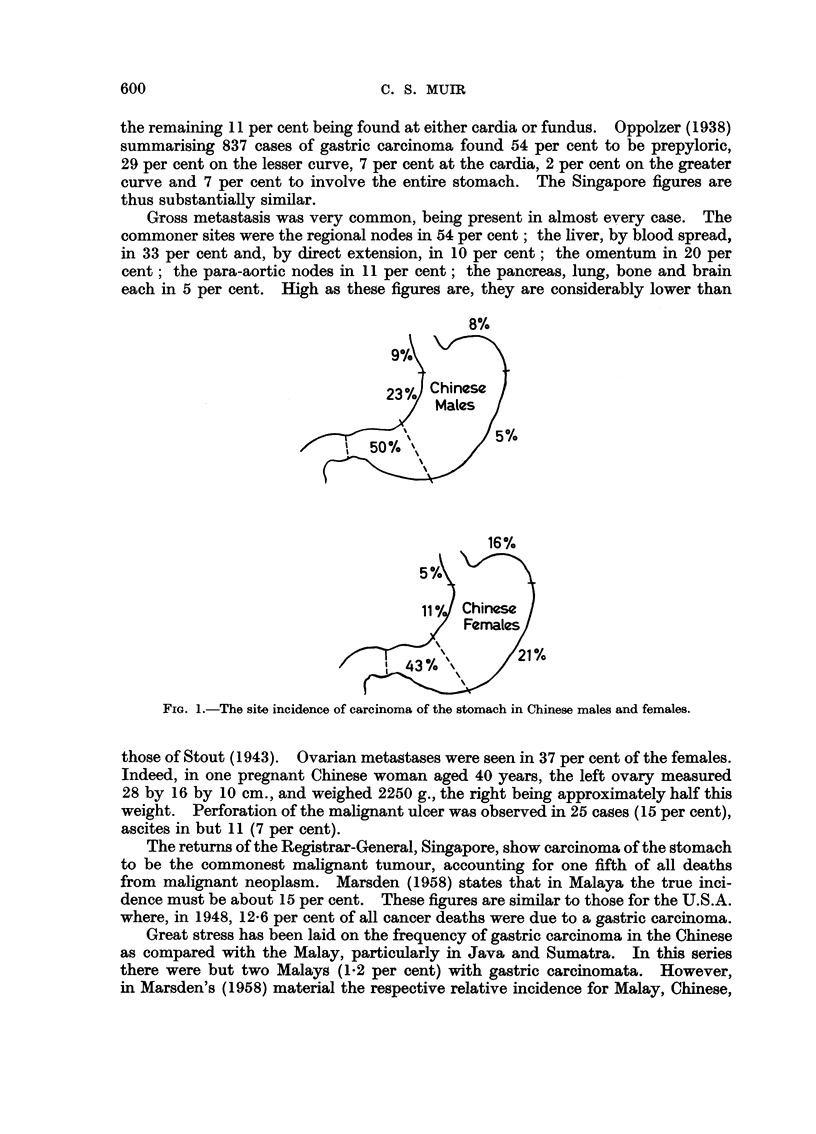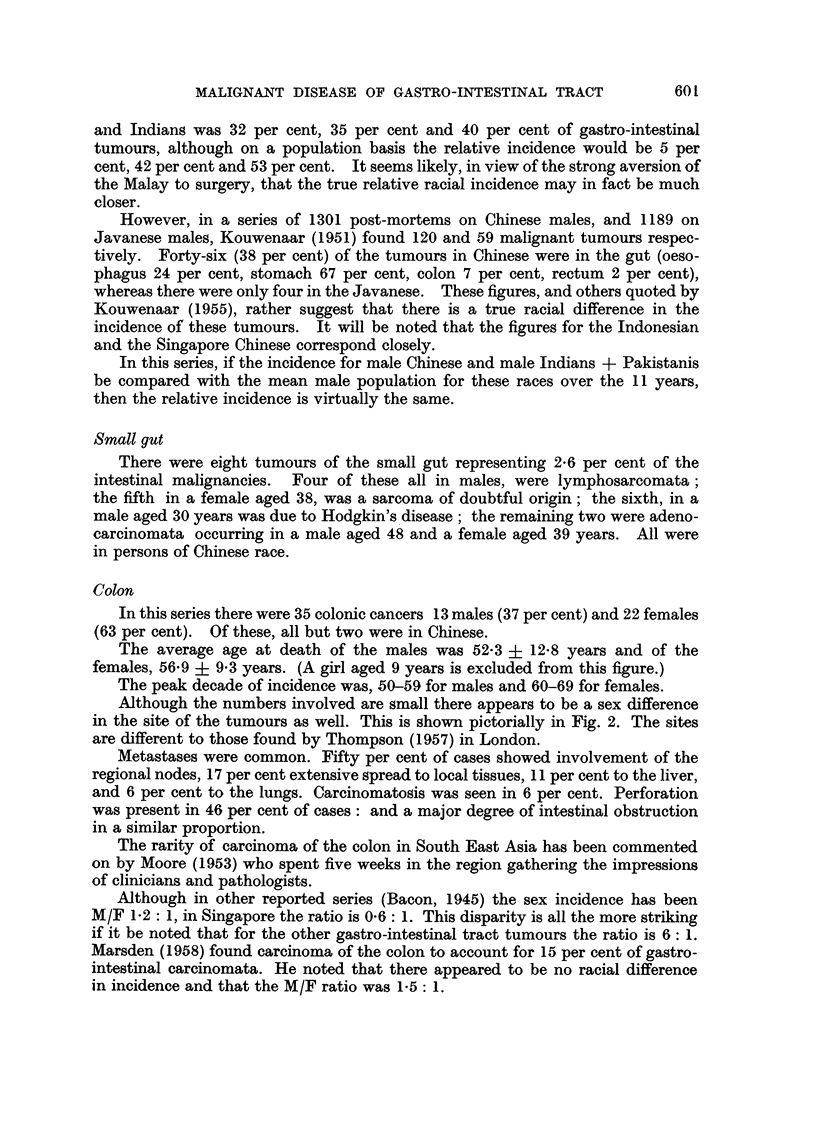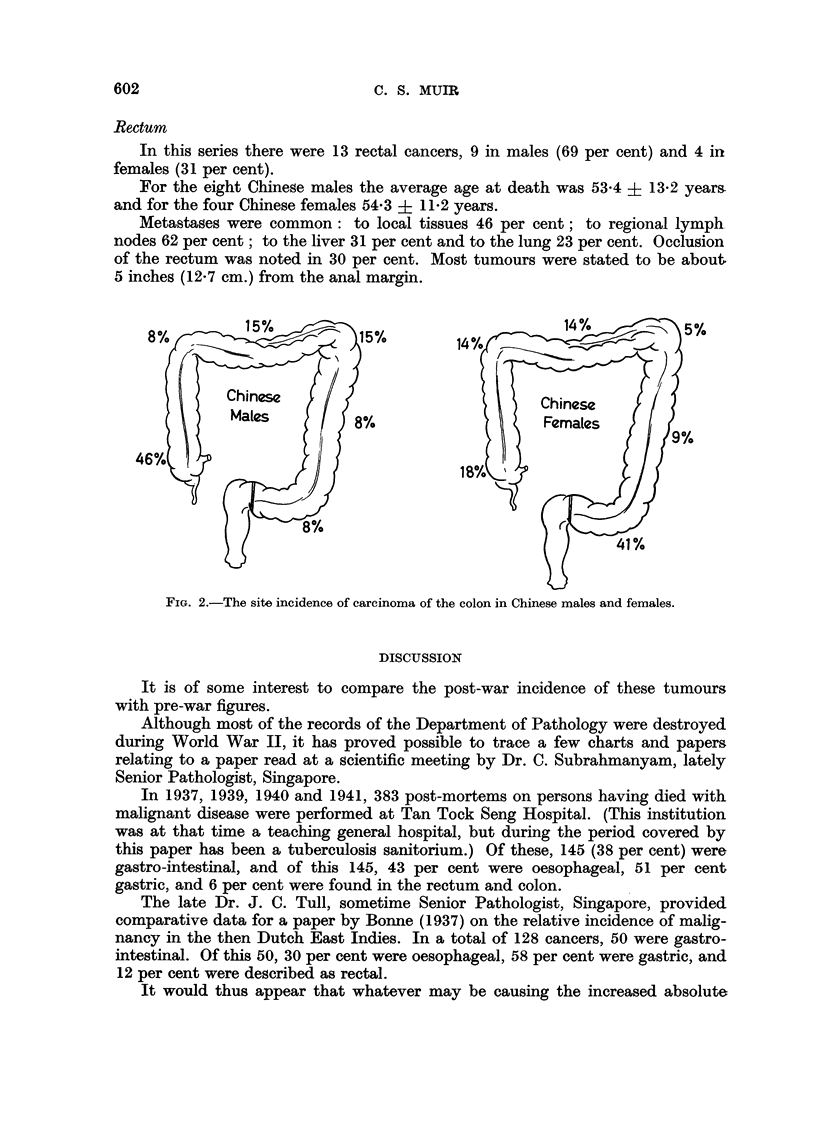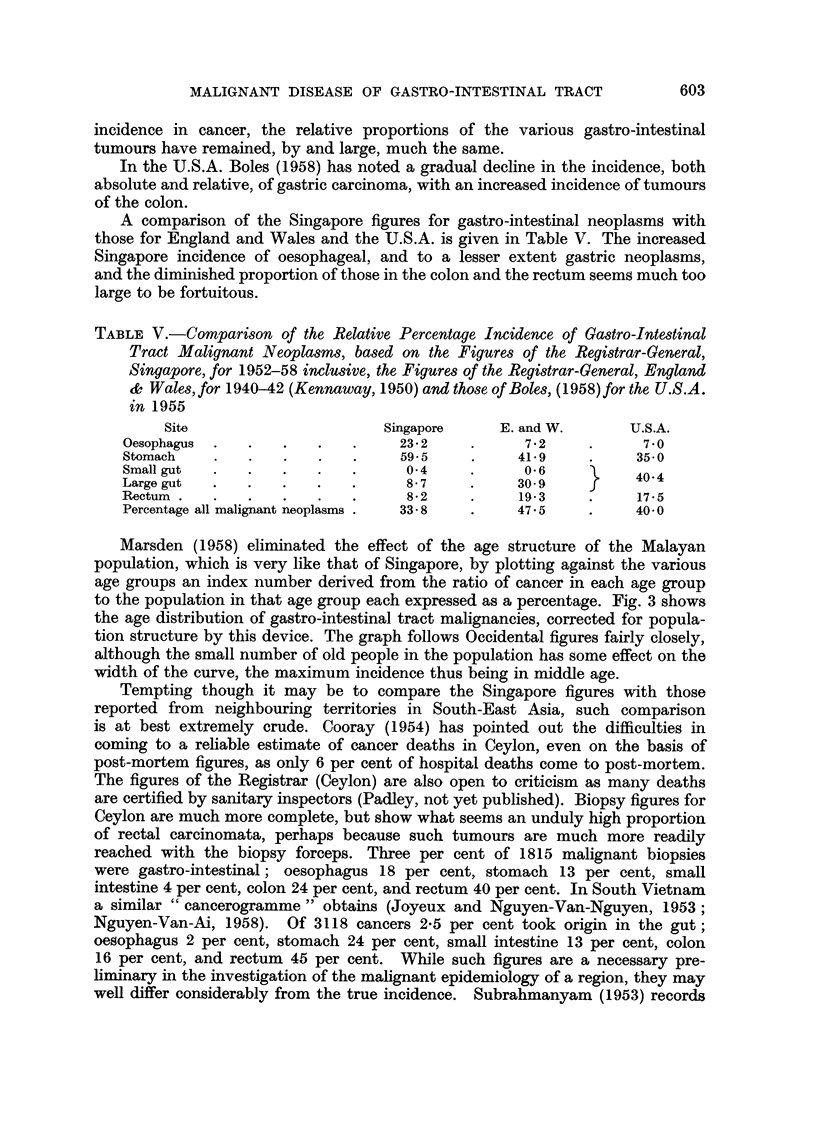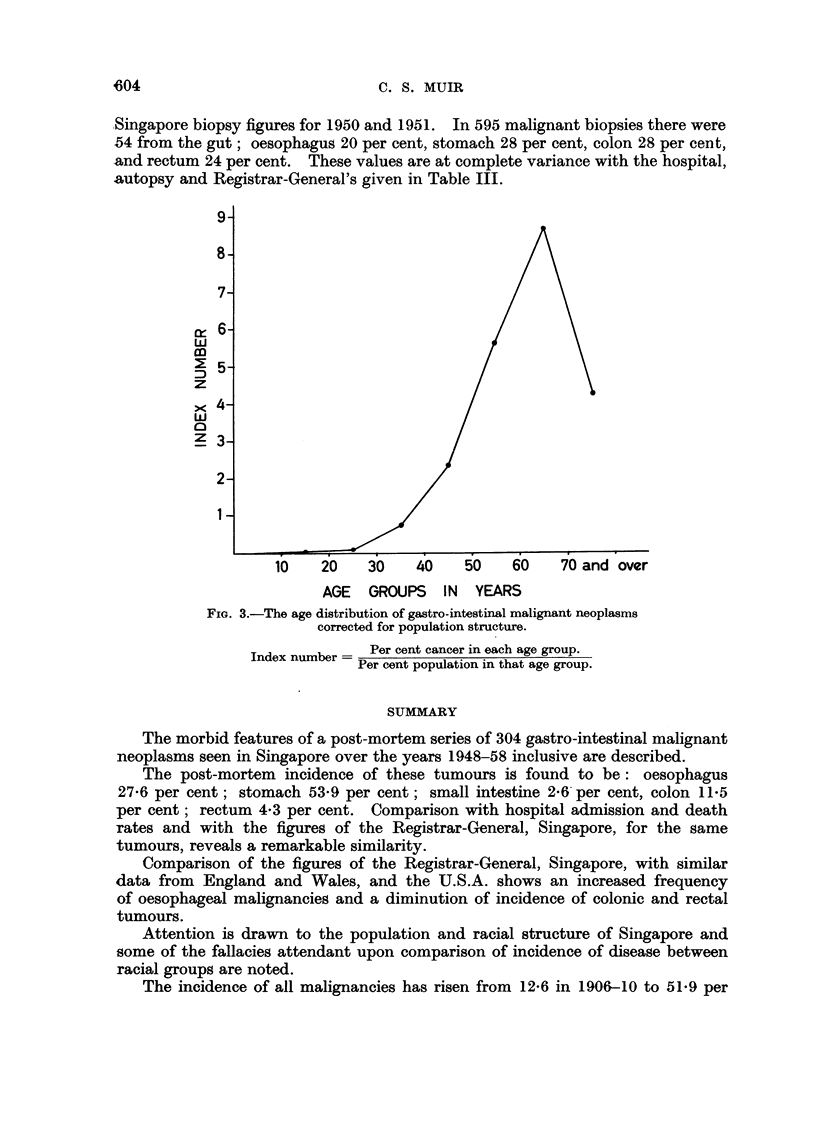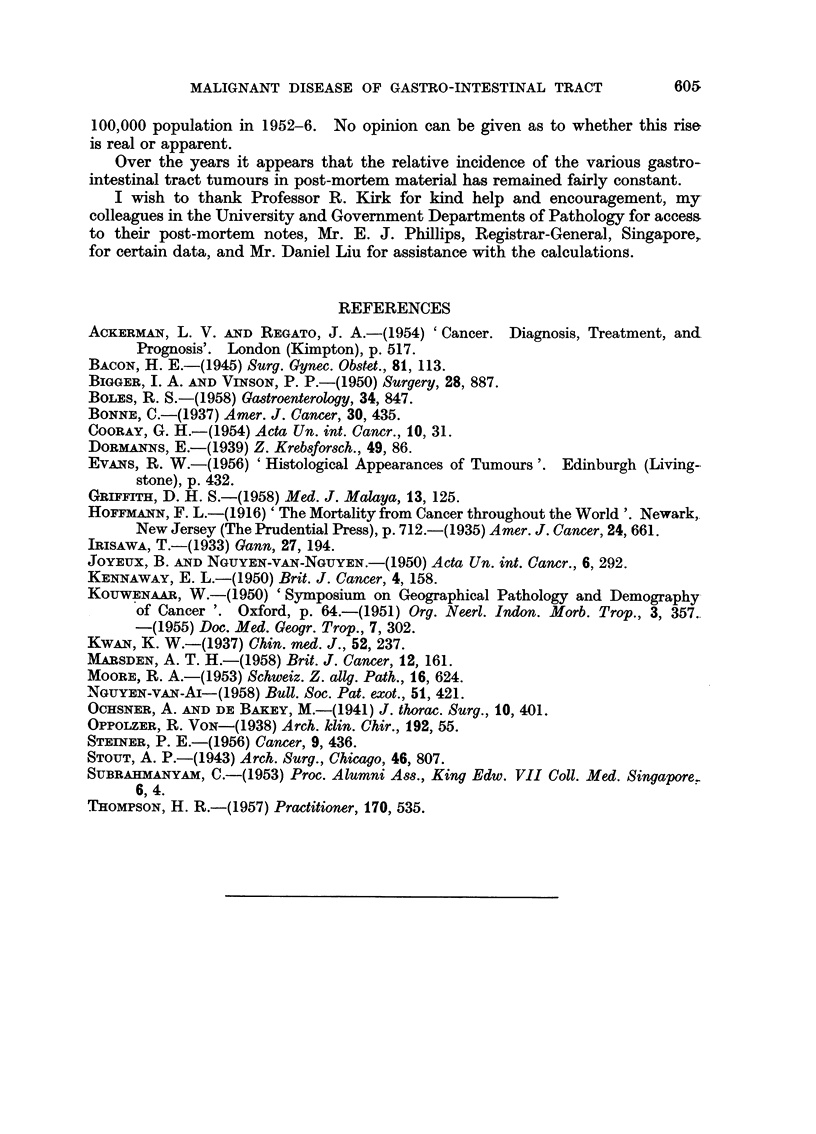# Malignant Disease of the Gastro-intestinal Tract in Singapore

**DOI:** 10.1038/bjc.1959.64

**Published:** 1959-12

**Authors:** C. S. Muir


### MALIGNANT DISEASE OF THE GASTRO-INTESTINAL TRACT IN SINGAPORE

C. S. MUIR

*From the Department of Pathology, University of Malaya in Singapore*

Received for publication October 1, 1959

SINGAPORE is singularly suitable, as is neighbouring Malaya, for the study of what has become known as Geographical Pathology. The presence of a mixed population of some one and a half millions, 75 per cent Chinese, 14 per cent Malay, and 9 per cent Indian and Pakistani, within an island of some 217 square miles, makes for an epidemiological unit of convenient size, all the more so as there is but one general hospital. Since the island lies one degree north of the equator, and one hundred and four degrees east of Greenwich, the climate is hot (average 85° F.), moist (average relative humidity 83 per cent), and equable. There are no seasons.

### METHODS : MATERIAL : SOURCES OF ERROR

At the mortuary of the General Hospital, Singapore, post-mortem examinations are carried out on persons who have died in the hospital, and on cases falling within the aegis of H.M. Coroner. In addition, a morbid anatomist from this department visits the Kandang Kerbau Maternity Hospital: post-mortem records are thus available from these three sources. A few post-mortem examinations carried out at a tuberculosis and a military hospital are not included. In short, records of almost all autopsies carried out in Singapore are available to this department.

All the post-mortem records of cases of malignant disease were scrutinised by the author and the data considered relevant were entered on a specially prepared pro forma for subsequent analysis.

This study covers the years 1948–58 inclusive, when 22,997 post-mortem examinations were carried out. In this material there were 304 deaths from malignant disease of the gastro-intestinal tract, i.e., 1·32 per cent. If the 11,525 children under the age of 11 years be excluded then the incidence is 2·65 per cent.

The age and sex incidence, by race, for the post-mortem population for the years 1950–58 inclusive is given in Table I, and the mean age and sex incidence, by race, for the entire population of Singapore for the period mid-1947 to mid-1957 is given in Table II. As most values have been approximated to the nearest round number some of the cross-additions may be apparently incorrect.

The sex disparity, once very great, in the population has been almost corrected since the cessation of unrestricted immigration. However, it will be noted that there is still an undue preponderance of Indian males in the third and subsequent decades.

Unfortunately, it has proved impossible to compile much of the data in this paper for the whole of the period covered by the post-mortem survey, and only such figures as were available have been presented.

TABLE I.—*The Age and Sex Distribution of the Singapore Post-Mortem Population, 1950–58 Inclusive*

| Age | Chinese Male | Chinese Female | Malay Male | Malay Female | Indian Male | Indian Female | Others Male | Others Female | Total Male | Total Female | Grand total Number | Grand total Per cent |
|---|---|---|---|---|---|---|---|---|---|---|---|---|
| 0–10 | 5,150 | 3,980 | 75 | 43 | 165 | 117 | 32 | 20 | 5,422 | 4,160 | 9,582 | 49·6 |
| 11–20 | 555 | 274 | 45 | 15 | 32 | 25 | 14 | 0 | 646 | 314 | 960 | 5·0 |
| 21–30 | 685 | 353 | 73 | 27 | 160 | 36 | 58 | 13 | 976 | 429 | 1,405 | 7·3 |
| 31–40 | 844 | 366 | 59 | 14 | 295 | 50 | 56 | 9 | 1,254 | 439 | 1,693 | 8·8 |
| 41–50 | 1,382 | 404 | 52 | 12 | 326 | 23 | 55 | 14 | 1,815 | 453 | 2,268 | 11·7 |
| 51–60 | 1,365 | 278 | 45 | 10 | 256 | 22 | 58 | 6 | 1,724 | 316 | 2,040 | 10·6 |
| 61–70 | 713 | 191 | 6 | 3 | 81 | 13 | 25 | 5 | 825 | 212 | 1,037 | 5·4 |
| 71+ | 191 | 95 | 10 | 7 | 15 | 4 | 9 | 5 | 225 | 111 | 336 | 1·7 |
| Totals | 10,885 | 5,941 | 365 | 131 | 1,330 | 290 | 307 | 72 | 12,887 | 6,434 | | |
| | 16,826 (87·0%) | | 496 (2·6%) | | 1,620 (8·4%) | | 379 (2·0%) | | 19,321 | | 19,321 | 100·1 |

TABLE II.—*Mean Age and Sex Distribution of the Singapore Population from Mid-1947 to Mid 1957*

| Age | Chinese Male | Chinese Female | Malaysians Male | Malaysians Female | Indians Male | Indians Female | Others Male | Others Female | Total Male | Total Female | Grand total Number | Grand total Per cent |
|---|---|---|---|---|---|---|---|---|---|---|---|---|
| 0–9 | 143,940 | 133,398 | 25,478 | 25,707 | 11,187 | 11,004 | 3,718 | 3,631 | 184,323 | 173,739 | 358,061 | 30·0 |
| 10–19 | 97,985 | 89,156 | 14,149 | 14,754 | 7,266 | 5,365 | 2,020 | 2,234 | 121,419 | 111,509 | 232,927 | 19·5 |
| 20–29 | 69,194 | 66,466 | 18,222 | 14,262 | 15,518 | 5,426 | 2,982 | 2,702 | 105,917 | 88,855 | 194,772 | 16·3 |
| 30–39 | 59,800 | 54,546 | 12,477 | 8,780 | 17,966 | 3,226 | 3,074 | 2,562 | 93,317 | 69,113 | 162,431 | 13·6 |
| 40–49 | 53,054 | 45,015 | 7,103 | 5,063 | 10,934 | 1,562 | 2,160 | 1,561 | 73,250 | 53,202 | 126,453 | 10·6 |
| 50–59 | 32,162 | 28,270 | 3,725 | 2,580 | 4,597 | 703 | 1,193 | 935 | 41,677 | 32,489 | 74,166 | 6·2 |
| 60–69 | 12,388 | 15,074 | 1,436 | 1,254 | 1,101 | 272 | 435 | 453 | 15,360 | 17,054 | 32,414 | 2·7 |
| 70+ | 3,209 | 6,636 | 635 | 739 | 273 | 113 | 149 | 237 | 4,266 | 7,726 | 11,992 | 1·0 |
| Totals | 471,732 | 438,561 | 83,225 | 73,139 | 68,842 | 27,671 | 15,731 | 14,315 | 639,529 | 553,687 | | |
| | 910,293 (76·3%) | | 156,364 (13·1%) | | 96,513 (8·1%) | | 30,046 (2·5%) | | 1,193,216 | | 1,193,216 | 99·9 |

It will be seen that there is considerable disparity between the post-mortem rates for the various races, the relative racial proportions for the population as a whole being Chinese 76·3 per cent, Malay 13·1 per cent, Indians 8·1 per cent, and Others 2·5 per cent, while the corresponding figures for post-mortems are 87·0, 2·6, 8·4 and 2·0 per cent.

Hence, unless the disease process under study is likely to cause sudden death, when an autopsy would be carried out irrespective of race at the mandate of H.M. Coroner, any post-mortem series will be quite unrepresentative racially, as for religious and other reasons Malays virtually never give consent for autopsy. Thus great care must be taken to ensure that the figures are comparable before concluding that any given neoplasm or disease process is rare or common in a particular racial group. Further, the requirement for autopsy in cases of sudden death in a racial group which is averse to morbid examination will give a false impression of the importance as a cause of death of, say, coronary heart disease.

The age structure of the Singapore population tends to give a falsely low impression of the relative incidence of malignant disease, just over half the population being under 21 years of age (1957). Further, the rate of natural increase is such that by 1962 half the population may well be under 15 years.

Over the years 1906–10, when the mean population was about 250,000 the cancer mortality rate per 100,000 population was 12·6 (Hoffman, 1916). For the period 1926–31, when the population was approximately double, the incidence was 40·6 (Hoffman, 1935). In 1957 the overall incidence was 53·2, and for the preceding five years the mean rate was 51·9. This gradual rise and maintenance of the rate of incidence at a time of explosive natural increase suggests either that the incidence of cancer as a whole is rising, or that the public is making better use of the improved medical facilities.

As post-mortem figures of incidence, both absolute and relative, are usually held to be biased, it was felt that no fair comment could be made unless hospital admissions, hospital deaths and the returns of the Registrar-General, Singapore, gave roughly comparable figures. That they do so tally to a remarkable degree

TABLE III.—*Comparison of the Number and Relative Incidence of Hospital Admissions, Hospital Deaths, Post-mortems, and Deaths recorded by the Registrar-General, Singapore, for Malignant Tumours of the Gastro-Intestinal Tract,* 1954–58 *Inclusive*

| International List No. | | Hospital admissions | | Hospital deaths | | Post-mortems | | Registrar-General | |
|---|---|---|---|---|---|---|---|---|---|
| | | Number | Per cent | Number | Per cent | Number | Per cent | Number | Per cent |
| 150 | Oesophagus | 485 | 27 | 172 | 34 | 48 | 32 | 299 | 24 |
| 151 | Stomach | 856 | 48 | 253 | 50 | 87 | 58 | 712 | 58 |
| 152 | Small gut | 12 | 1 | 1 | — | 2 | 1 | 2 | — |
| 153 | Large gut | 179 | 10 | 43 | 8 | 7 | 5 | 112 | 9 |
| 154 | Rectum | 241 | 14 | 39 | 8 | 6 | 4 | 109 | 9 |

is shown in Table III. As the system of classification of the returns of both the hospital and the Registrar-General has altered several times in the past ten years it was not possible to complete this table for the period covered by the post-mortem survey.

The figures of the Registrar-General, Singapore, are probably the most accurate in South-East Asia as 55 per cent of deaths are certified by doctors, 12·5 per cent by H.M. Coroner, usually following a post-mortem examination, and 32·5 per cent by Police Officers and Hospital Assistants (1957). In neighbouring Malaya, in 1956, 80·7 per cent of deaths were not certified by a medical practitioner. The majority of deaths certified by unqualified persons are ascribed to " sawan " (convulsions) or " demum " (fever), (Griffith, 1958). Neoplasia is rarely, if ever, mentioned.

Neoplasms of the gastro-intestinal tract form a high proportion of all malignant tumours. In Table IV the incidence of these tumours, as recorded by the Registrar-General, Singapore, is given, and expressed as a percentage of the 3,777 malignant neoplasms recorded in the same period.

TABLE IV.—*Incidence of Gastro-Intestinal Tract Neoplasms Recorded by the Registrar-General, Singapore,* 1952–57 *Inclusive*

| Site | Number | Per cent | Percentage of all malignant neoplasms, i.e. of 3777 |
|---|---|---|---|
| Oesophagus | 283 | 22·0 | 7·5 |
| Stomach | 783 | 61·0 | 20·7 |
| Small intestine | 6 | 0·5 | 0·2 |
| Colon | 109 | 8·5 | 2·9 |
| Rectum | 103 | 8·0 | 2·7 |
| Total | 1284 | 100·0 | 34·0 |

*The oesophagus*

There were 84 cases of oesophageal carcinoma, 73 males (87 per cent) and 11 females. The mean age at death for the 11 females, who were all Chinese, was 56·1 $\pm$ 13·4 years, and for the 69 Chinese males 53·9 $\pm$ 9·4 years, 2 Indian Hindus, 1 Indian Muslim and 1 Malay brought the male total to 73.

The peak incidence for males was in the decades 40–49 and 50–59, while for the females it was in the decades 50–59 and 60 –69.

Ochsner and de Bakey (1941) who collected 8572 cases of oesophageal carcinoma from the medical literature found that 20 per cent were situated in the upper-third, 37 per cent in the middle third, and 43 per cent in the lower third. Comparable figures for this series are 6 per cent, 50 per cent and 40 per cent. The site was not stated in the remaining 4 per cent.

The majority of the carcinomata were described as being about 10 cm. in diameter : obstruction was noted in 72 per cent.

Fistulae were common, occurring in 40 per cent of upper third, 38 per cent of middle third, and 10 per cent of lower third carcinomata. Those in the upper third communicated with the trachea ; those in the middle third with the lung direct, the bronchi, the trachea and the pericardium in that order ; those of the lower third with the lung direct and the pericardium.

Not unexpectedly, abscess formation was common. Fifteen per cent of unoperated cases showed a concomitant lung abscess, 5 per cent a mediastinal abscess and 2 per cent a brain abscess.

Metastatic spread was extremely common. The mediastinal glands were grossly involved in 45 per cent of cases, the abdominal glands in 18 per cent and the cervical glands in 11 per cent. There was spread to local tissues in 30 per cent, to the lung in 13 per cent, to the liver and brain in 10 per cent of cases. These figures are in rough agreement with those of Dormanns (1939) who analysed the sites of metastasis in 824 cases of oesophageal carcinoma.

Oesophageal carcinomata seem to be much commoner in Singapore than in the Occident, forming 27·6 per cent of this series, and about 8 per cent of all neoplastic deaths (Table IV).

Ackerman and Regato (1954) state that in the U.S.A. approximately 2 per cent of all deaths from carcinoma are oesophageal.

Oesophageal growths have long been known to be very common in China. Kwan (1937) found about half of gastro-intestinal growths were in the oesophagus, and a similar high incidence in the Chinese of Java has been noted (Kouwenaar, 1950).

Marsden (1958), in an analysis of biopsy material in Malaya, found carcinoma of the oesophagus in 19 per cent, 25 per cent and 17 per cent of gastro-intestinal tumours in Malays, Chinese, and Indians respectively.

Much stress has been laid on the consumption of hot food as an aetiological factor and, as the Chinese male was reputed to be the first in the family to eat from the dish, the male preponderance was held to be accounted for. This, as Marsden (1958) comments, is certainly not true for either Malaya or Singapore, and he suggests that the drinking of spirits is a much more likely cause. The relatively high incidence of oesophageal cancer in Japan has also been attributed to alcohol (Irisawa, 1933) and Steiner (1956) discusses this relationship at length. Despite the frequency with which the Chinese female in Singapore attempts suicide by drinking caustic soda, no history of such an episode was recorded in the 11 females, although in other reports the association has been noted (Bigger and Vinson, 1950).

*The stomach*

There were 164 cases of carcinoma of the stomach. Of these 143 (87 per cent) were male, and 21 (13 per cent) female.

One hundred and forty-seven were of Chinese race. Of these 128 were male, and 19 female. The mean age at death for the males was 51·7 $\pm$ 10·5 years and for the females 45·9 $\pm$ 11·7 years.

The peak incidence was found to be in the decades 40–49 and 50–59 for the male (in all, 67 per cent), and the decades 30–39 and 50 –59 for the female (respectively 37 per cent and 32 per cent).

There were 13 cases in Indians, of whom 12 were male. The mean age at death for the males was 48·2 $\pm$ 11·5 years, the peak incidence being in the decade 50–59.

The sites of the tumours are given in Fig. 1. It will be noted that there is some sex disparity in the incidence of greater curve tumours. As the number of females is small, the significance of this finding is doubtful. Nevertheless an interesting point is raised which may repay further study.

Evans (1956) finds that approximately 65 per cent of carcinomata are in the prepyloric region, 20 per cent on the lesser curve, and 4 per cent on the greater, the remaining 11 per cent being found at either cardia or fundus. Oppolzer (1938) summarising 837 cases of gastric carcinoma found 54 per cent to be prepyloric, 29 per cent on the lesser curve, 7 per cent at the cardia, 2 per cent on the greater curve and 7 per cent to involve the entire stomach. The Singapore figures are thus substantially similar.

Gross metastasis was very common, being present in almost every case. The commoner sites were the regional nodes in 54 per cent ; the liver, by blood spread, in 33 per cent and, by direct extension, in 10 per cent ; the omentum in 20 per cent ; the para-aortic nodes in 11 per cent ; the pancreas, lung, bone and brain each in 5 per cent. High as these figures are, they are considerably lower than

FIG. 1.—The site incidence of carcinoma of the stomach in Chinese males and females.

those of Stout (1943). Ovarian metastases were seen in 37 per cent of the females. Indeed, in one pregnant Chinese woman aged 40 years, the left ovary measured 28 by 16 by 10 cm., and weighed 2250 g., the right being approximately half this weight. Perforation of the malignant ulcer was observed in 25 cases (15 per cent), ascites in but 11 (7 per cent).

The returns of the Registrar-General, Singapore, show carcinoma of the stomach to be the commonest malignant tumour, accounting for one fifth of all deaths from malignant neoplasm. Marsden (1958) states that in Malaya the true incidence must be about 15 per cent. These figures are similar to those for the U.S.A. where, in 1948, 12·6 per cent of all cancer deaths were due to a gastric carcinoma.

Great stress has been laid on the frequency of gastric carcinoma in the Chinese as compared with the Malay, particularly in Java and Sumatra. In this series there were but two Malays (1·2 per cent) with gastric carcinomata. However, in Marsden's (1958) material the respective relative incidence for Malay, Chinese, and Indians was 32 per cent, 35 per cent and 40 per cent of gastro-intestinal tumours, although on a population basis the relative incidence would be 5 per cent, 42 per cent and 53 per cent. It seems likely, in view of the strong aversion of the Malay to surgery, that the true relative racial incidence may in fact be much closer.

However, in a series of 1301 post-mortems on Chinese males, and 1189 on Javanese males, Kouwenaar (1951) found 120 and 59 malignant tumours respectively. Forty-six (38 per cent) of the tumours in Chinese were in the gut (oesophagus 24 per cent, stomach 67 per cent, colon 7 per cent, rectum 2 per cent), whereas there were only four in the Javanese. These figures, and others quoted by Kouwenaar (1955), rather suggest that there is a true racial difference in the incidence of these tumours. It will be noted that the figures for the Indonesian and the Singapore Chinese correspond closely.

In this series, if the incidence for male Chinese and male Indians + Pakistanis be compared with the mean male population for these races over the 11 years, then the relative incidence is virtually the same.

*Small gut*

There were eight tumours of the small gut representing 2·6 per cent of the intestinal malignancies. Four of these all in males, were lymphosarcomata; the fifth in a female aged 38, was a sarcoma of doubtful origin; the sixth, in a male aged 30 years was due to Hodgkin's disease; the remaining two were adenocarcinomata occurring in a male aged 48 and a female aged 39 years. All were in persons of Chinese race.

*Colon*

In this series there were 35 colonic cancers 13 males (37 per cent) and 22 females (63 per cent). Of these, all but two were in Chinese.

The average age at death of the males was 52·3 $\pm$ 12·8 years and of the females, 56·9 $\pm$ 9·3 years. (A girl aged 9 years is excluded from this figure.)

The peak decade of incidence was, 50–59 for males and 60–69 for females.

Although the numbers involved are small there appears to be a sex difference in the site of the tumours as well. This is shown pictorially in Fig. 2. The sites are different to those found by Thompson (1957) in London.

Metastases were common. Fifty per cent of cases showed involvement of the regional nodes, 17 per cent extensive spread to local tissues, 11 per cent to the liver, and 6 per cent to the lungs. Carcinomatosis was seen in 6 per cent. Perforation was present in 46 per cent of cases: and a major degree of intestinal obstruction in a similar proportion.

The rarity of carcinoma of the colon in South East Asia has been commented on by Moore (1953) who spent five weeks in the region gathering the impressions of clinicians and pathologists.

Although in other reported series (Bacon, 1945) the sex incidence has been M/F 1·2 : 1, in Singapore the ratio is 0·6 : 1. This disparity is all the more striking if it be noted that for the other gastro-intestinal tract tumours the ratio is 6 : 1. Marsden (1958) found carcinoma of the colon to account for 15 per cent of gastro-intestinal carcinomata. He noted that there appeared to be no racial difference in incidence and that the M/F ratio was 1·5 : 1.

*Rectum*

In this series there were 13 rectal cancers, 9 in males (69 per cent) and 4 in females (31 per cent).

For the eight Chinese males the average age at death was $53 \cdot 4 \pm 13 \cdot 2$ years and for the four Chinese females $54 \cdot 3 \pm 11 \cdot 2$ years.

Metastases were common : to local tissues 46 per cent ; to regional lymph nodes 62 per cent ; to the liver 31 per cent and to the lung 23 per cent. Occlusion of the rectum was noted in 30 per cent. Most tumours were stated to be about 5 inches (12·7 cm.) from the anal margin.

FIG. 2.—The site incidence of carcinoma of the colon in Chinese males and females.

DISCUSSION

It is of some interest to compare the post-war incidence of these tumours with pre-war figures.

Although most of the records of the Department of Pathology were destroyed during World War II, it has proved possible to trace a few charts and papers relating to a paper read at a scientific meeting by Dr. C. Subrahmanyam, lately Senior Pathologist, Singapore.

In 1937, 1939, 1940 and 1941, 383 post-mortems on persons having died with malignant disease were performed at Tan Tock Seng Hospital. (This institution was at that time a teaching general hospital, but during the period covered by this paper has been a tuberculosis sanitorium.) Of these, 145 (38 per cent) were gastro-intestinal, and of this 145, 43 per cent were oesophageal, 51 per cent gastric, and 6 per cent were found in the rectum and colon.

The late Dr. J. C. Tull, sometime Senior Pathologist, Singapore, provided comparative data for a paper by Bonne (1937) on the relative incidence of malignancy in the then Dutch East Indies. In a total of 128 cancers, 50 were gastro-intestinal. Of this 50, 30 per cent were oesophageal, 58 per cent were gastric, and 12 per cent were described as rectal.

It would thus appear that whatever may be causing the increased absolute incidence in cancer, the relative proportions of the various gastro-intestinal tumours have remained, by and large, much the same.

In the U.S.A. Boles (1958) has noted a gradual decline in the incidence, both absolute and relative, of gastric carcinoma, with an increased incidence of tumours of the colon.

A comparison of the Singapore figures for gastro-intestinal neoplasms with those for England and Wales and the U.S.A. is given in Table V. The increased Singapore incidence of oesophageal, and to a lesser extent gastric neoplasms, and the diminished proportion of those in the colon and the rectum seems much too large to be fortuitous.

TABLE V.—*Comparison of the Relative Percentage Incidence of Gastro-Intestinal Tract Malignant Neoplasms, based on the Figures of the Registrar-General, Singapore, for* 1952–58 *inclusive, the Figures of the Registrar-General, England & Wales, for* 1940–42 (*Kennaway,* 1950) *and those of Boles,* (1958) *for the U.S.A. in* 1955

| Site | Singapore | E. and W. | U.S.A. |
|---|---|---|---|
| Oesophagus | 23·2 | 7·2 | 7·0 |
| Stomach | 59·5 | 41·9 | 35·0 |
| Small gut | 0·4 | 0·6 | 40·4 (small and large gut) |
| Large gut | 8·7 | 30·9 | |
| Rectum | 8·2 | 19·3 | 17·5 |
| Percentage all malignant neoplasms | 33·8 | 47·5 | 40·0 |

Marsden (1958) eliminated the effect of the age structure of the Malayan population, which is very like that of Singapore, by plotting against the various age groups an index number derived from the ratio of cancer in each age group to the population in that age group each expressed as a percentage. Fig. 3 shows the age distribution of gastro-intestinal tract malignancies, corrected for population structure by this device. The graph follows Occidental figures fairly closely, although the small number of old people in the population has some effect on the width of the curve, the maximum incidence thus being in middle age.

Tempting though it may be to compare the Singapore figures with those reported from neighbouring territories in South-East Asia, such comparison is at best extremely crude. Cooray (1954) has pointed out the difficulties in coming to a reliable estimate of cancer deaths in Ceylon, even on the basis of post-mortem figures, as only 6 per cent of hospital deaths come to post-mortem. The figures of the Registrar (Ceylon) are also open to criticism as many deaths are certified by sanitary inspectors (Padley, not yet published). Biopsy figures for Ceylon are much more complete, but show what seems an unduly high proportion of rectal carcinomata, perhaps because such tumours are much more readily reached with the biopsy forceps. Three per cent of 1815 malignant biopsies were gastro-intestinal; oesophagus 18 per cent, stomach 13 per cent, small intestine 4 per cent, colon 24 per cent, and rectum 40 per cent. In South Vietnam a similar "cancerogramme" obtains (Joyeux and Nguyen-Van-Nguyen, 1953; Nguyen-Van-Ai, 1958). Of 3118 cancers 2·5 per cent took origin in the gut; oesophagus 2 per cent, stomach 24 per cent, small intestine 13 per cent, colon 16 per cent, and rectum 45 per cent. While such figures are a necessary preliminary in the investigation of the malignant epidemiology of a region, they may well differ considerably from the true incidence. Subrahmanyam (1953) records

Singapore biopsy figures for 1950 and 1951. In 595 malignant biopsies there were 54 from the gut; oesophagus 20 per cent, stomach 28 per cent, colon 28 per cent, and rectum 24 per cent. These values are at complete variance with the hospital, autopsy and Registrar-General's given in Table III.

FIG. 3.—The age distribution of gastro-intestinal malignant neoplasms corrected for population structure.

$$\text{Index number} = \frac{\text{Per cent cancer in each age group.}}{\text{Per cent population in that age group.}}$$

SUMMARY

The morbid features of a post-mortem series of 304 gastro-intestinal malignant neoplasms seen in Singapore over the years 1948–58 inclusive are described.

The post-mortem incidence of these tumours is found to be: oesophagus 27·6 per cent; stomach 53·9 per cent; small intestine 2·6 per cent, colon 11·5 per cent; rectum 4·3 per cent. Comparison with hospital admission and death rates and with the figures of the Registrar-General, Singapore, for the same tumours, reveals a remarkable similarity.

Comparison of the figures of the Registrar-General, Singapore, with similar data from England and Wales, and the U.S.A. shows an increased frequency of oesophageal malignancies and a diminution of incidence of colonic and rectal tumours.

Attention is drawn to the population and racial structure of Singapore and some of the fallacies attendant upon comparison of incidence of disease between racial groups are noted.

The incidence of all malignancies has risen from 12·6 in 1906–10 to 51·9 per

100,000 population in 1952–6. No opinion can be given as to whether this rise is real or apparent.

Over the years it appears that the relative incidence of the various gastro-intestinal tract tumours in post-mortem material has remained fairly constant.

I wish to thank Professor R. Kirk for kind help and encouragement, my colleagues in the University and Government Departments of Pathology for access to their post-mortem notes, Mr. E. J. Phillips, Registrar-General, Singapore, for certain data, and Mr. Daniel Liu for assistance with the calculations.

### REFERENCES

ACKERMAN, L. V. AND REGATO, J. A.—(1954) 'Cancer. Diagnosis, Treatment, and Prognosis'. London (Kimpton), p. 517.
BACON, H. E.—(1945) *Surg. Gynec. Obstet.*, **81**, 113.
BIGGER, I. A. AND VINSON, P. P.—(1950) *Surgery*, **28**, 887.
BOLES, R. S.—(1958) *Gastroenterology*, **34**, 847.
BONNE, C.—(1937) *Amer. J. Cancer*, **30**, 435.
COORAY, G. H.—(1954) *Acta Un. int. Cancr.*, **10**, 31.
DORMANNS, E.—(1939) *Z. Krebsforsch.*, **49**, 86.
EVANS, R. W.—(1956) 'Histological Appearances of Tumours'. Edinburgh (Livingstone), p. 432.
GRIFFITH, D. H. S.—(1958) *Med. J. Malaya*, **13**, 125.
HOFFMANN, F. L.—(1916) 'The Mortality from Cancer throughout the World'. Newark, New Jersey (The Prudential Press), p. 712.—(1935) *Amer. J. Cancer*, **24**, 661.
IRISAWA, T.—(1933) *Gann*, **27**, 194.
JOYEUX, B. AND NGUYEN-VAN-NGUYEN.—(1950) *Acta Un. int. Cancr.*, **6**, 292.
KENNAWAY, E. L.—(1950) *Brit. J. Cancer*, **4**, 158.
KOUWENAAR, W.—(1950) 'Symposium on Geographical Pathology and Demography of Cancer'. Oxford, p. 64.—(1951) *Org. Neerl. Indon. Morb. Trop.*, **3**, 357.—(1955) *Doc. Med. Geogr. Trop.*, **7**, 302.
KWAN, K. W.—(1937) *Chin. med. J.*, **52**, 237.
MARSDEN, A. T. H.—(1958) *Brit. J. Cancer*, **12**, 161.
MOORE, R. A.—(1953) *Schweiz. Z. allg. Path.*, **16**, 624.
NGUYEN-VAN-AI—(1958) *Bull. Soc. Pat. exot.*, **51**, 421.
OCHSNER, A. AND DE BAKEY, M.—(1941) *J. thorac. Surg.*, **10**, 401.
OPPOLZER, R. VON—(1938) *Arch. klin. Chir.*, **192**, 55.
STEINER, P. E.—(1956) *Cancer*, **9**, 436.
STOUT, A. P.—(1943) *Arch. Surg., Chicago*, **46**, 807.
SUBRAHMANYAM, C.—(1953) *Proc. Alumni Ass., King Edw. VII Coll. Med. Singapore*, **6**, 4.
THOMPSON, H. R.—(1957) *Practitioner*, **170**, 535.